# Sequence Characterization of Extra-Chromosomal Circular DNA Content in Multiple Blackgrass (*Alopecurus myosuroides*) Populations

**DOI:** 10.3390/genes14101905

**Published:** 2023-10-04

**Authors:** Wangfang Fu, Dana R. MacGregor, David Comont, Christopher A. Saski

**Affiliations:** 1 Department of Plant and Environmental Sciences, Clemson University, Clemson, SC 29634, USA; wfu@g.clemson.edu; 2 Rothamsted Research, Protecting Crops and the Environment, Harpenden, Hertfordshire AL5 2JQ, UK; dana.macgregor@rothamsted.ac.uk (D.R.M.); david.comont@rothamsted.ac.uk (D.C.)

**Keywords:** eccDNA, blackgrass, herbicide resistance, rapid adaptation

## Abstract

*Alopecurus myosuroides* (blackgrass) is a problematic weed of Western European winter wheat, and its success is largely due to widespread multiple-herbicide resistance. Previous analysis of F2 seed families derived from two distinct blackgrass populations exhibiting equivalent non-target site resistance (NTSR) phenotypes shows resistance is polygenic and evolves from standing genetic variation. Using a CIDER-seq pipeline, we show that herbicide-resistant (HR) and herbicide-sensitive (HS) F3 plants from these F2 seed families as well as the parent populations they were derived from carry extra-chromosomal circular DNA (eccDNA). We identify the similarities and differences in the coding structures within and between resistant and sensitive populations. Although the numbers and size of detected eccDNAs varied between the populations, comparisons between the HR and HS blackgrass populations identified shared and unique coding content, predicted genes, and functional protein domains. These include genes related to herbicide detoxification such as Cytochrome P450s, ATP-binding cassette transporters, and glutathione transferases including *AmGSTF1*. eccDNA content was mapped to the *A. myosuroides* reference genome, revealing genomic regions at the distal end of chromosome 5 and the near center of chromosomes 1 and 7 as regions with a high number of mapped eccDNA gene density. Mapping to 15 known herbicide-resistant QTL regions showed that the eccDNA coding sequences matched twelve, with four QTL matching HS coding sequences; only one region contained HR coding sequences. These findings establish that, like other pernicious weeds, blackgrass has eccDNAs that contain homologs of chromosomal genes, and these may contribute genetic heterogeneity and evolutionary innovation to rapidly adapt to abiotic stresses, including herbicide treatment.

## 1. Introduction

Extra-chromosomal circular DNAs (eccDNAs) are ring-shaped amplifications of linear genomic segments that exist separate from the autosomal genome. These structures have been observed in various species, including plant and higher eukaryote species [1,2,3,4,5,6]. Bassel and Hoota first discovered eccDNAs in 1964 [7], but limited progress in understanding their function or genesis was made until the past decade. The formation of eccDNA can occur through multiple mechanisms, including non-allelic homologous recombination, replication slippage and DNA repair [8,9], and intramolecular recombination and retrotransposon activity [1,10,11,12]. Historically, the detection of eccDNAs has been challenging due to their complex and highly repetitive structures. However, recent advancements in hybrid sequencing technologies (Illumina, PacBio, Oxford Nanopore) and computational algorithms (AmpliconArchitect [13], CiderSeq [14], and Circle-Seq [15]) have improved the ability to detect eccDNAs and define tissue-specific “DNA circulome” [13,16].

eccDNAs have recently garnered significant attention in scientific communities as a unique hallmark of somatic mutations. These entities are capable of amplifying gene copies, related transcripts, and regulatory elements. eccDNAs can range in size from a few hundred base pairs [1] to much larger structures near a megabase in size [12,17], with a majority being smaller than 10 kb [1,18]. eccDNAs accumulate in cells through either known mechanisms of autonomous replication mediated by a replication origin [11,12,19,20], rolling circle amplification [21], DNA replication and mitosis [22,23], or some elusive mechanism. Notably, the accumulation of many replicate copies of a large eccDNA in Palmer amaranth has been shown to significantly increase the c-value of cells, seemingly without fitness penalty [24].

eccDNAs have been identified in various human physiological and pathological states, including normal cells (blood and muscle) [25], the central nervous system and neurodegeneration [26], aging and telomere maintenance [27,28], and cancer malignancy and progression [16]. eccDNAs have been observed in Drosophilia [4], and are believed to play a role in stress adaptation in yeast [29,30]. eccDNAs have also been found in plants. For instance, Nipponbare rice has seventeen copies of the long terminal repeat retrotransposon (LTR-RT) *PopRice* and, of these, eleven produce ecDNA [31]. *PopRice* is important for regulating the seed-to-seedling transition through altering the balance of hormone signaling in the endosperm [32]. More specifically, endosperm-specific expression is induced by gibberellins, and they sequester abscisic-acid (ABA)-related transcription factors such as Rice ABA INSENSITVE5 (OsABI5) [32]. eccDNAs have also been found in mutant Arabidopsis plants affected in DNA methylation and post-transcriptional gene silencing [33]. These were derived from transposable elements and led to changes in genome instability and the accumulation of structural variations probably acting through DNA-repair pathways [33].

Most relevantly, eccDNAs have been shown to play a role in herbicide resistance in weeds [19]. The eccDNAs present in the noxious weed Palmer amaranth have been well-characterized. This eccDNA, which is the largest gene-containing eccDNA reported thus far in a plant species, confers genetically transmissible resistance to the herbicide glyphosate [12,19]. This eccDNA replicon has multiple copies of the gene encoding for 5-enolpyruvylshikimate-3-phosphate (EPSP) synthase (EPSPS) and serves as the basis for gene copy amplification and, therefore, resistance to the EPSPS-inhibiting herbicide glyphosate. A study comparing eccDNA from glyphosate-sensitive and -resistant populations revealed a significant content of eccDNA in glyphosate-sensitive populations [12]. The EPSPS gene was only present on eccDNA in glyphosate-resistant populations, while glyphosate-sensitive eccDNA displayed the genomic disposition for recombination events that may lead to the formation of the eccDNA replicon [1]. High-resolution cytological analysis of the eccDNA replicon revealed that its persistence in the germline is facilitated through a chromosomal tethering mechanism rather than genomic re-integration [19]. This observation provides an explanation for the uneven segregation reported in previous studies [19]. Sequence analysis of the eccDNA replicon in Palmer amaranth revealed that it contains 58 genes in addition to the EPSPS gene, as well as a complex distribution of repeat sequences and gene functions that encompass detoxification, transport, replication, and stress response [12]. A synteny analysis of the other genes aligned with a neighboring species revealed that they originated from multiple chromosomes, suggesting a complex biogenesis event [12]. This study suggests that eccDNAs serve as a rapid response mechanism and facilitate rapid adaptation [1].

Blackgrass (*Alopecurus myosuroides*) is a pernicious weed that has rapidly evolved to infest cereal crops across Northern and Western Europe and is now widely regarded as the top weed problem in several regions [34,35]. In response to the extensive use of herbicides, blackgrass has evolved resistance to seven herbicide modes-of-action, exacerbating this species’ impact [36]. Blackgrass has been estimated to cause an annual GBP 0.4 billion lost gross profit in the UK alone, due to wheat yield losses of 0.8 million tonnes [37]. Growers are now increasingly relying upon mixtures and sequences of (particularly pre-emergent) herbicides from a range of modes-of-action to manage this species, alongside physical or cultural practices. Nevertheless, further herbicidal control is complicated by the extensive presence of both gene mutations and multiple mechanisms that inactivate herbicides in this species [38], evolving independently within separate populations [39].

Advancements in comprehending the genetic basis of herbicide resistance have been limited. Nevertheless, recent studies have uncovered the genome of blackgrass, revealing independent and semi-overlapping genetic mechanisms underlying non-target site resistance (NTSR) in blackgrass at both the DNA and RNA levels [39]. To date, the presence and potential contribution of eccDNA to herbicide resistance in populations of *A. myosuroides* has not been considered, however. Here, using the same genetic material as a prior study of the genetic basis of NTSR in this species [39], we perform sequence characterization of extra-chromosomal circular DNA (eccDNA) content in multiple blackgrass populations with well-characterized levels of heritable NTSR-based herbicide resistance. We compare the genomic similarities and differences among individuals from these distinct populations. Additionally, we determine the gene content of the eccDNA and identify genomic hotspots for eccDNA biogenesis, evaluating their role in the rapid evolution of herbicide resistance.

## 2. Materials and Methods

### 2.1. Blackgrass Populations, Plant Material, and Genomic DNA Extraction

Seven blackgrass seed populations were grown for extraction of eccDNA, representing both herbicide-sensitive (HS) and herbicide-resistant (HR) lines. The Roth (HS) population originates from the Rothamsted ‘Broadbalk’ long-term field experiment and represents a wild-type population which has never experienced herbicide. The ‘Peldon’ and ‘Lola91’ are field-collected HR populations confirmed to have non-target-site resistance to a range of herbicide chemistries. For these seed populations, we have selected against any known target-site mutations [40]. Further HR and HS samples were derived from segregating families created through biparental crossing of Roth and Peldon (CC2) and Roth and Lola 91 (CC5). The CC2R and CC2S are F_3_ generation lines, caused by identifying and bulk-crossing the most and least resistant CC2 individuals within the F_2_ generation. The CC5R and CC5S were produced in an identical way, but from the CC5 seed family. In total, this provides three herbicide-sensitive (Roth, CC2S, CC5S) populations and four herbicide-resistant (Peldon, Lola91, CC2R, CC5R) populations with which to examine eccDNA.

Seeds from these seven populations were broadcast sown onto a standard seed tray (37.3 × 24.5 × 5.5 cm) filled with Rothamsted Prescription Mix (75% Medium grade (L&P) peat, 12% Screened sterilized loam, 3% Medium grade vermiculite, 10% Grit (5 mm screened, lime free, with 3.5 kg Osmocote Exact 3/4 month per m3—Supplier: Scotts UK Professional, Ipswich, Suffolk, UK). Plants were grown in standard glasshouse (GH43 101 t) at 10 °C/5 °C 16 h day length without supplementary lighting for 40 days (between 15 December 2021 and 25 January 2022) until they were at the 2–3 leaf stage. From each population, two bulk harvests of aerial tissue from these plants totaling 0.5 g were cut, flash frozen in liquid nitrogen, and ground completely to a fine powder in a mortar and pestle. A total of 100 mg of this ground material was then used for DNA extraction.

DNA was extracted from 100 mg of finely ground flash-frozen bulked leaf material from 40-day-old seedlings using a modified CTAB method [41]. Briefly, DNA was extracted from flash-frozen leaf material that had been ground in liquid nitrogen using 2× CTAB buffer with β-mercaptoethanol and then separated away from the proteins and other cellular components that partition into the organic phase using a chloroform wash. DNA was then precipitated from the aqueous phase using cold isopropanol, and the pellet was washed with 70% ethanol. The cleaned DNA pellet was dried using a speedvac before being resuspended in TE buffer with RNase A Solution. Samples were quantified via nanodrop analysis, then stored at −20 °C until being shipped on dry ice for eccDNA enrichment and sequencing.

### 2.2. eccDNA Sequence Enrichment, Sequencing, and Analysis

Circular DNA enrichment and sequencing was performed according to the CIDER-seq methods [14]. Circular DNA sequencing was performed on a Pacific Biosciences Sequel II instrument (Pacific Biosciences) with a 30 h movie time and HiFi/CCS read generation. Sequence reads were deconcatenated into circular DNA using the CIDER-seq software [14]. The circular DNA sequences were then aligned to the reference genome of *A. myosuroides* [14] to predict the extra-chromosomal circular DNA (eccDNA) content. To reduce redundant sequences, the eccDNA sequences were clustered and collapsed into non-redundant representatives using the CD-hit program [42] with a sequence identity threshold of 0.9. The representative eccDNA sequences were masked for repetitive elements using the Repeat-Masker [43] with default settings and annotated using the MAKER annotation pipeline [44].

GO enrichment analysis was conducted on the predicted coding elements of the eccDNA using the GOseq 1.42.0 R package [45]. GO terms were considered significantly enriched at a False Discovery Rate (FDR) of less than 0.05. The enriched GO terms were visualized using the ggplot2 package [46]. tRNAs were exclusively predicted for the eccDNA sequences without coding sequences (CDS) using the tRNAscan-SE program [47] with default settings. To determine the genomic origin of the eccDNA, the predicted sequences were first aligned to the reference genome using the Minimap2 program [48]. The number of eccDNAs that overlapped with the reference genome in each 500 kb interval was counted using the BEDtools software [49]. The number of genes and transposable elements (TEs) were also counted within 200 kb downstream and upstream of each gene using BEDtools [49]. A permutation test was performed to compare the mean number of genes and TEs between the eccDNA-present and -absent regions.

## 3. Results

### 3.1. eccDNA Content and Coding Structure in Multiple Blackgrass Populations

**Figure 1:**
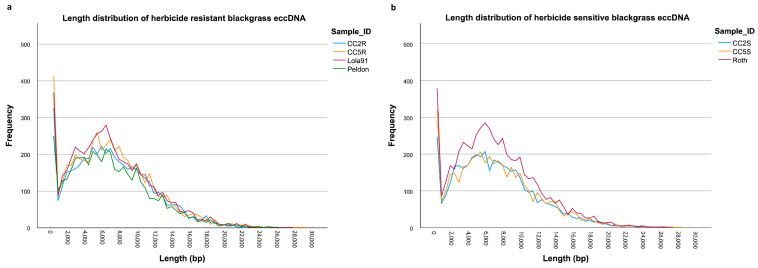
eccDNA length distribution in herbicide-resistant (**a**) and -sensitive (**b**) blackgrass populations.

The CIDER-seq pipeline was used to identify a high abundance of extra-chromosomal circular DNA (eccDNA) in all blackgrass samples (Table 1). The number of non-redundant representative eccDNAs detected in the blackgrass samples ranged from 4233 in sample CC2S to 5663 in sample Roth, with an average of 4886 (Table 1). The size of the detected eccDNAs showed high variation within the samples, as evidenced by the length distributions, which ranged from 31 bp to 29,081 bp and had mean lengths of around 6900 bp (Table 1 and Figure 1). The length distributions of the eccDNAs were similar among all sequenced samples (Table 1 and Figure 1).

We further analyzed the coding content of the predicted eccDNA sequences. The number of eccDNAs with predicted genes ranged from 958 in sample CC5S to 1291 in sample Roth (average of 1079 per sample) and accounted for up to approximately 22% of the total predicted eccDNAs (Table 1). The number of predicted genes per eccDNA ranged from 1 to 15, with an average of 2 genes per eccDNA across all analyzed samples (Tables S1 and S2). Transfer RNA (tRNA) prediction was performed for the eccDNAs and revealed a relatively low number of predicted tRNAs in all samples, ranging from 45 in sample CC5S to 77 in sample CC2S.

### 3.2. Coding Content of eccDNAs in Herbicide-Resistant and -Sensitive Blackgrass Populations

**Figure 2:**
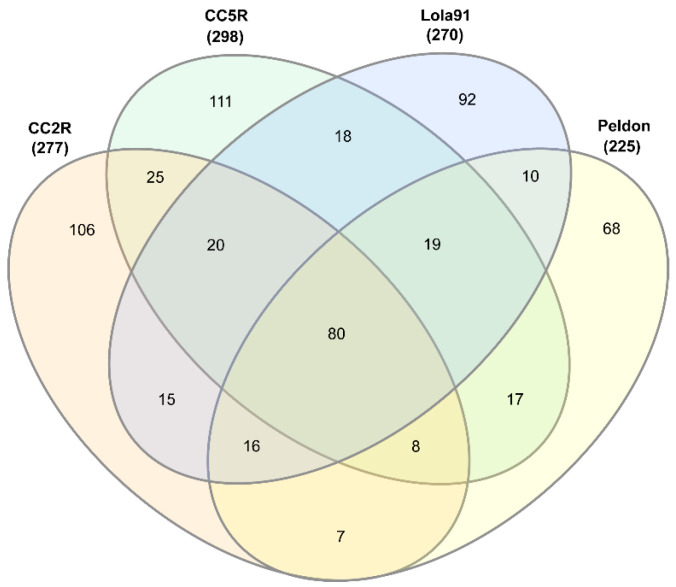
Venn diagram of PFAM elements shared by herbicide-resistant eccDNA samples.

**Figure 3:**
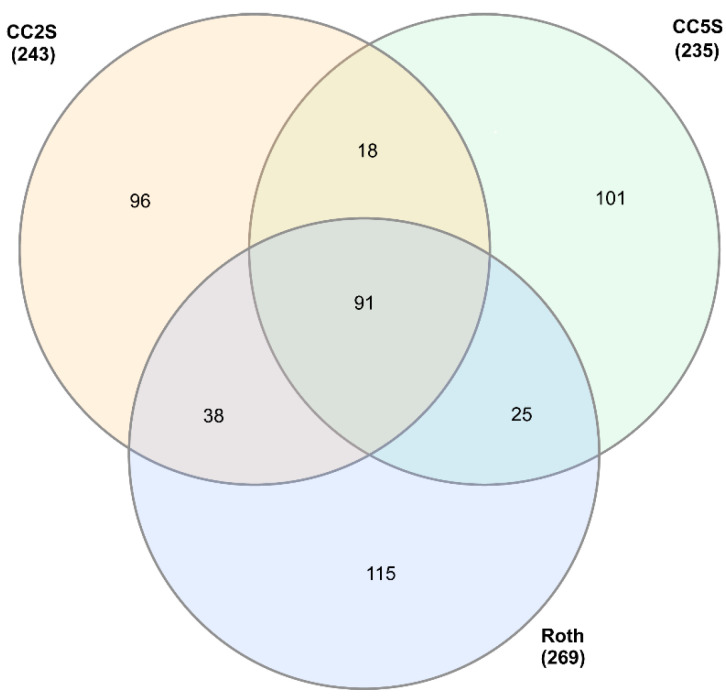
Venn diagram of PFAM elements shared by herbicide-sensitive eccDNA samples.

To identify common and unique eccDNA-encoded genes with predicted functional protein domains among different blackgrass populations, we compared the predicted gene content from both herbicide-resistant (HR) (CC2R, CC5R, Lola91, and Peldon) and -sensitive (HS) (CC2S, CC5S, and Roth) blackgrass samples (Tables S1 and S2). A comparison of the predicted protein domains (PFAM id) identified a total of 80 protein coding domains that were shared by all HR samples (Figure 2 and Table S3). Among these functional protein domains, the number of eccDNAs annotated to them ranged from 6 to 416. There was a range of 7 to 25 functional annotations that were shared by at least two HR samples. The different HR samples had varying numbers of unique functional protein domains, ranging from 68 (Peldon) to 111 (CC5R) (Table S3). Overall, the three HS samples shared a total of 91 functional protein domains (Figure 3 and Table S4). Pairwise comparisons between the HS samples revealed that 18 to 38 protein domains were shared by each of the two samples. In addition, 96 (CC2S) to 115 (Roth) unique functional domains were identified in different HS samples. By comparing the 91 functional protein domains shared by the three HS samples to the 80 protein coding domains shared by all four HR samples, a list of 69 functional protein domains that are common to all measured samples was obtained. Additionally, there are 11 functional protein domains specific to all the HR samples and 22 functional protein domains specific to all the HS samples (Table S5). Counts of these core sets of functional protein domains found in all samples range from 416 to 6 in the HR samples and 5 in the HS samples and include putative gypsy type transposons, Proton-conducting membrane transporters, ribosomal protein domains, and domains associated with the respiratory chain, such as NADH-ubiquinone/plastoquinone oxidoreductase or NADH dehydrogenase, and the photosynthetic machinery, such as Photosystem I psaA/psaB protein and Photosystem II protein (Table S5). Counts for the functional protein domains that were unique to either HR (14 to 6) or HS (15 to 3) were comparatively low and are discussed below.

In the HR samples, the functional domains with the highest abundance comprised proteins associated with photosystems, gypsy type transposons, NADH dehydrogenase, PPR repeat, and Cytochrome C assembly protein (Table 2). Moreover, we observed the presence of eccDNA genes in the analyzed HR samples, which were previously reported to be associated with herbicide detoxification. Cytochrome P450 was detected in all HR samples, whereas ATP-binding cassette transporters (ABC transporters) and glutathione transferases were exclusively detected in the CC2R, CC5R, and Peldon populations (Table S3). Furthermore, within the HR samples, various stress response domains were identified. Specifically, ribosomal proteins and leucine-rich repeats were present in all HR samples. The WRKY DNA-binding domain was shared among the CC2R, CC5R, and Lola91 samples. Moreover, the Myb-like DNA-binding domain and WD domain were shared by the CC5R, Lola91, and Peldon samples. Finally, peroxidase was detected in the CC2R, Lola91, and Peldon samples.

The functional domains detected in herbicide-sensitive (HS) samples were found to be abundant and largely overlapping with those detected in herbicide-resistant (HR) samples, as demonstrated in Table 2, Table 3 and Table S3. Three functional domains, namely the Jacalin-like lectin domain, the GDSL/SGNH-like Acyl-Esterase family, and the PMR5 N-terminal domain, were present exclusively in the three HS samples. Additionally, annotations related to herbicide detoxification were identified in the HS samples, including the presence of cytochrome P450 and glutathione transferases in all HS samples and an ABC transporter annotated only to predicted eccDNA genes in CC5S and Roth (Supplementary Table S4).

**Figure 4:**
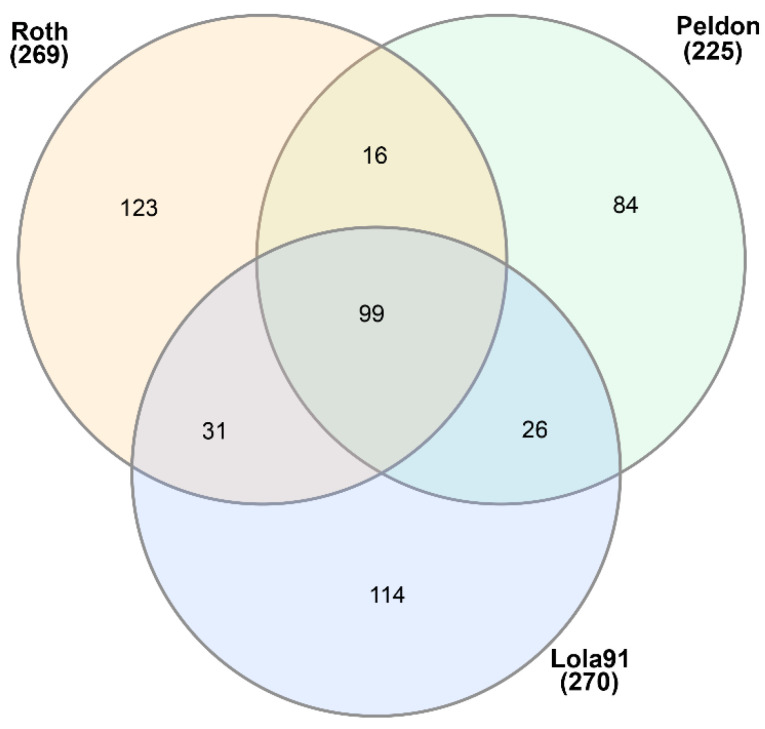
Venn diagram of PFAM elements shared by parents of the herbicide sensitive and resistant populations in this study.

**Figure 5:**
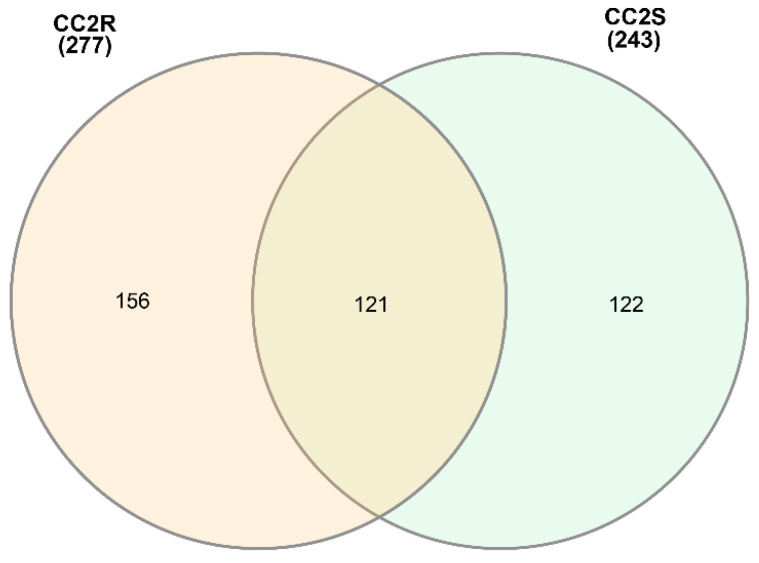
Venn diagram of PFAM elements shared by CC2 population.

**Figure 6:**
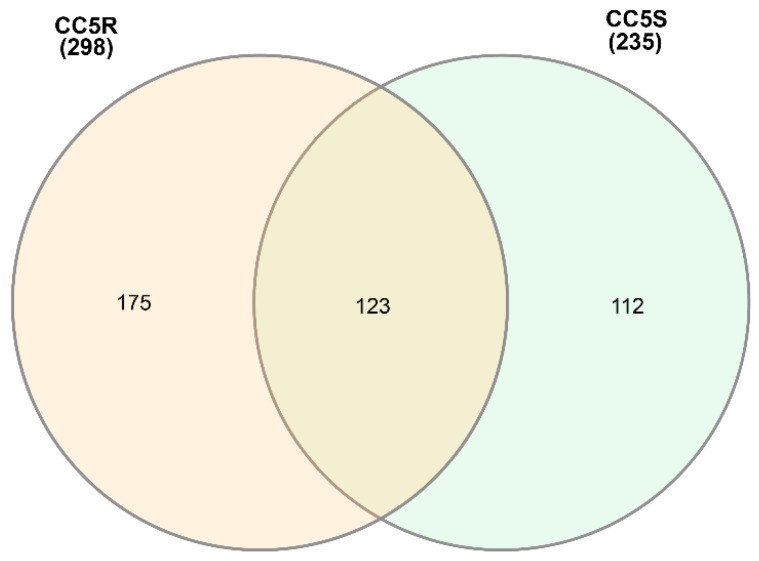
Venn diagram of PFAM elements shared by CC5 population (herbicide resistant and susceptible).

To better understand the inheritance of eccDNA in blackgrass, we further compared the functional domains separately in the parents (Figure 4), CC2 (Figure 5), and CC5 (Figure 6, Supplementary Tables S6–S8) populations. In total, 99 functional domains were shared by the three parents, and 26 functional domains were shared by the two HR parents (Peldon and Lola91), while 123 functional domains were only detected in the HS parent (Roth) (Figure 4 and Table S6). Some defense-related functional domains, such as the DnaJ domain, glycosyl hydrolase, and response regulator receiver domains, were uniquely identified in the HR parents.

Upon comparing the two distinct biotypes within the CC2 population, we found that functional annotations associated with ABC transporters were exclusively identified in the CC2R (HR) samples (Table S7). In contrast, within the CC5 population, functional annotations linked to ABC transporters were detected in both the CC5R and CC5S samples.

To gain insights into the functions of the predicted extra-chromosomal circular DNA (eccDNA) genes, we conducted a homology-based analysis with previously identified blackgrass herbicide-resistant candidate genes [50]. Our results showed that eccDNAs homologous to the crucial herbicide-resistant gene, GSTF1 (ALOMY3G11302 [39]), were detected exclusively in the herbicide-resistant (HR) samples CC2R and CC5R (Table 4). Of particular interest is that this sequence is 39 amino acids shorter than the other four gene sequences previously identified [51], but otherwise most closely aligns with the GST2c sequence. ALOMY3G11302 was differentially expressed in both CC2R and CC5R populations, as reported by [39]. eccDNAs homologous to GSTU2 were present in both HR and HS samples. The presence of eccDNAs homologous to OPR1 and GSTF2 was restricted to Peldon and CC5S, respectively. We also found sequences that associated with PF00662 (NADH-Ubiquinone oxidoreductase), common to all the samples (Supplementary Table S5), as well as those associated with PF01370, the NAD-dependent epimerase/dehydratase in the CC5R, Peldon, and Roth samples (Supplementary Table S1).

### 3.3. Gene Ontology Enrichment of Blackgrass eccDNA

**Figure 7:**
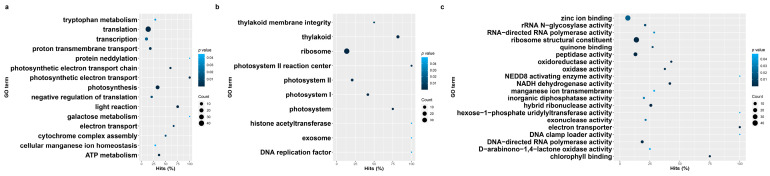
Gene ontology enrichment terms and their prevalence among herbicide-resistant blackgrass eccDNA samples. (**a**) Biological process, (**b**) cellular components, (**c**) molecular functions.

Gene ontology (GO) enrichment analysis of predicted coding contents in eccDNA samples showed a list of GO terms associated with biological processes, cellular components, and molecular function that were enriched in HR and HS samples. In HR samples, the enriched biological processes include translation, transcription, protein neddylation, photosynthesis, cytochrome complex assembly, ATP-related processes, and galactose metabolic processes (Figure 7a and Supplementary Table S9). For the cellular component category, GO terms that related to the photosystem, thylakoid, and ribosome were enriched in HR samples (Figure 7b and Supplementary Table S9). Representative molecular functions for HR eccDNAs include zinc ion binding, UDP-glucose, structure constituents of ribosome, and chlorophyll binding (Figure 7c and Supplementary Table S9).

**Figure 8:**
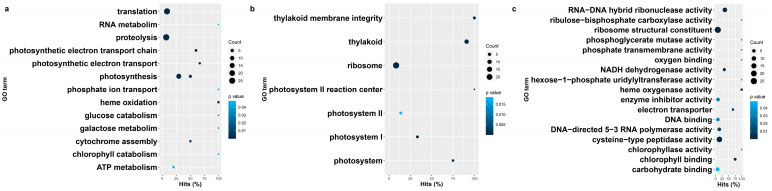
Gene ontology enrichment terms and their prevalence among herbicide-sensitive blackgrass eccDNA samples. (**a**) Biological process, (**b**) cellular components, (**c**) molecular functions.

Compared with the HR samples, similar but slightly different GO terms were enriched in HS samples. Biological processes such as the chlorophyll catabolic process, glucose catabolic process, RNA metabolic process, heme oxidation, phosphate ion transport, and proteolysis were only enriched in HS samples (Figure 7a and Figure 8a and Supplementary Tables S9 and S10), while biological process such as ATP synthesis, protein neddylation, transcription, tryptophan metabolic processes, and cellular manganese ion homeostasis were uniquely enriched in HR samples. All GO terms related to cellular components that were enriched in HR samples were also enriched in HS samples except for the DNA replication factor, exosome, and H4/H2A histone acetyltransferase complex (Figure 7b and Figure 8b and Supplementary Tables S9 and S10). Molecular functions such as chlorophyllase activity, enzyme inhibitor activity, phosphoglycerate mutase activity, and double-stranded DNA binding were only identified in HS samples (Figure 7c and Figure 8c and Supplementary Tables S9 and S10), while GO terms related to oxidoreductase activity, ADP binding, exonuclease activity, and manganese ion transmembrane transporter activity were uniquely enriched in HR samples.

### 3.4. Genomic Origins of Blackgrass eccDNA

**Figure 9:**
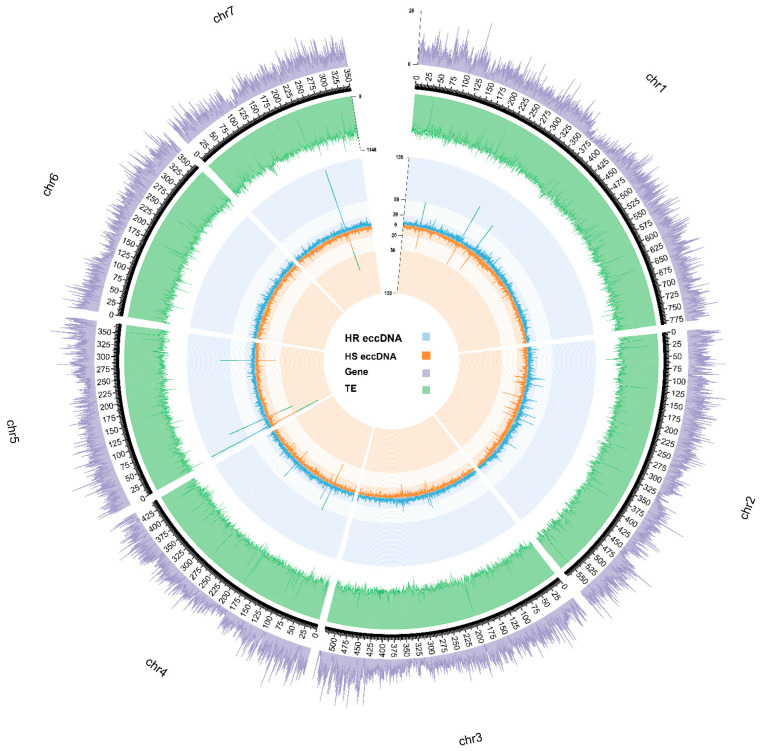
Circos plot showing the count of eccDNAs identified in herbicide-resistant (blue) and -sensitive (orange) blackgrass population. Outer ring shows the number of genes (purple) and transposable elements (green) in the reference genome.

To determine the genomic origins of eccDNA, the predicted eccDNAs were mapped to the *A. myosuroides* reference genome [39]. The numbers of mapped eccDNAs were counted within the non-overlapped windows of 500 kb through the whole genome (Figure 6 and Figure 9, and Supplementary Table S11). Over 90% of the detected eccDNAs were successfully mapped to the established chromosomes, and around 8.5% of the predicted eccDNAs were mapped to the unanchored sequences. Several genomic segments were identified, with a high frequency of eccDNAs being mapped to these regions (Figure 9). These regions include the distal end of chromosome 5 and the near center of chromosomes 1 and 7 (Figure 6 and Table 5). The 500 kb window localized at the near center of chromosome 1 contained 83 eccDNAs from HR samples and 48 eccDNAs from HS samples. The two 500 kb windows located at the distal end of chromosome 5 contained 237 (HR 133, HS 104) and 137 (HR 82, HS 55) predicted eccDNAs, respectively. And the 500 kb window on chromosome 7 mapped 130 and 80 eccDNAs from HS and HR samples, respectively. Stress-related genes, such as the ribosomal family, lysine decarboxylase, and HPPK, were located in these regions. However, no eccDNA coding contents were mapped to these regions (Figure 9).

In order to gain further insight into the genomic features of the regions enriched with eccDNA-associated genes, we analyzed whether the eccDNA-associated genes originated from regions with a higher gene density than expected by chance (Figure 6). We observed that eccDNA-present regions tend to have higher number of genes in comparison with eccDNA-absent regions in both HR and HS samples. In both HR and HS samples, we observed significant differences (permutation test for difference of means, *n* = 10,000 replicates) between the number of genes located in eccDNA-present regions and eccDNA-absent regions. The mean number of observed genes in eccDNA-present regions was increased by 0.24 (*p* = 0.0019) and 0.26 (*p* = 0.0044) in comparison with eccDNA-absent regions in HR and HS samples, respectively. Compared with eccDNA-absent regions, eccDNA-present regions tend to have less TE (HR = 3.16, HS = 1.37); however, the difference in the number of TEs in eccDNA-present regions and -absent regions was only significant in HR samples (*p* = 0.026). Notably, no significant differences were observed between the number of observed genes or TE in eccDNA-present regions and eccDNA-absent regions when all samples were analyzed together.

A prior study identified a list of quantitative trait loci (QTL) regions that are associated with herbicide resistance in various blackgrass populations [39]. To investigate the mapping of eccDNAs to these regions, we found that 496 HR eccDNAs and 352 HS eccDNAs were mapped to the blackgrass herbicide-resistant QTL regions (Supplementary Table S11). Our analysis revealed that within the QTL regions, two non-overlapping windows of 500 kb located on chromosome 3 (230.0–230.5 Mb and 312.5–313.0 Mb) were uniquely mapped to at least three of the HR samples (Supplementary Table S11). eccDNA coding sequences were mapped to 12 QTL regions (Supplementary Table S12, where the qtl-cc2-2-1, qtl-cc2-2-3, qtl-cc2-5-1, and qtl-cc2-5-2 regions only contained HS eccDNA coding sequences (CDS), while the qtl-cc5-3-3 region only contained HR eccDNA CDS. Additionally, we observed that among the eccDNA CDS mapped to the QTL regions, only HR eccDNA genes were successfully annotated with functional predictions. Most of these genes were predicted to be related to the photosystem and gypsy type transposons (Supplementary Table S12).

## 4. Discussion

Extra-chromosomal circular DNAs (eccDNAs) are DNA molecules that are separate from the main chromatin body, or chromosomes, within a cell and are gaining significant attention with their role in trait biology [1,5,9,12,16,20,27]. This form of DNA has been demonstrated to play a crucial role in various physiological processes. One of the most prominent functions of eccDNA is its capacity to harbor and amplify segments of transcriptionally active chromatin, resulting in gene focal amplifications. Furthermore, eccDNA acts as a reservoir of genetic diversity that the cell can draw upon during exposure to stress, whether it be biotic or abiotic, facilitating rapid responses and evolutionary adaptations [1,52]. The extensive use of herbicides over the years has led to the evolution of blackgrass populations that are resistant to multiple herbicides, including those from different chemical classes [38]. Aside from the rapid evolution of herbicide resistance, adaptive traits, such as a lifecycle that occurs within a standard winter-cropping time, high fecundity, rapid growth, and the ability to compete with the crop for nutrients, light, and water, have enabled blackgrass to thrive in agricultural landscapes across Northern and Western Europe and become a major challenge for these farmers.

In this research, we have uncovered a wide array of eccDNAs within several blackgrass populations, showcasing varying levels of herbicide resistance. Our findings in blackgrass, concerning size, prevalence, and coding content, align closely with *Amaranthus palmeri* [1]. In general, we identified a suite of encoded genes and transposable elements associated with signaling, transport, and DNA mobility in both herbicide-resistant and -sensitive populations, indicating a possible role in adaptability. For example, a number of *gypsy* retroelements were found on eccDNA which have been previously implicated with a role in creating genetic diversity [53]. We identified a significant number of proton-conducting membrane transporter genes (Table S3) which function in the regulation of proton movement across cell membranes and have been associated with enabling plants to modulate their responses to various environmental stressors such as salinity, drought, and metal toxicity, which enhance their adaptability [54,55]. We also found an abundance of NADH dehydrogenase functional domains generally in the eccDNA coding dataset. This gene class is part of the mitochondrial electron transport chain and is involved in energy production, redox balance, and respiratory metabolism. Because of the previous identification of the role of eccDNAs in glyphosate resistance we looked for the presence of shikimate pathway genes in our eccDNA samples. We found the Pfam term PF01202, which is associated with Shikimate kinase in the CC5R samples (Tables S3 and S7). Its presence on an eccDNA may influence the expression amplitudes of these genes and contribute to the plant’s ability to cope with stress through the maintenance of cellular energy levels and redox homeostasis during adverse environmental conditions [56,57]. Ribosomal proteins were also highly abundant, indicating a need for altered protein turnover, which has also been reported in [1]. Transcription factors such as WRKY, MYB, and other classes (Table S3) were also generally found in the blackgrass populations on eccDNA. These classes of transcription factors are pivotal regulators in plants that enable the activation of key processes such as stress responses, defense mechanisms, and various developmental process which are critical in allowing plants to integrate these signals and fine-tune the adaptative potential [58,59,60]. The presence of these transcription factors on eccDNA indicates their relevance as rapid response elements.

A principal goal of our study was to examine the signatures of adaptation in the eccDNA content between blackgrass populations that have rapidly evolved metabolic herbicide resistance with those that have not. Interestingly, gene ontology enrichment analysis between the contrasting populations did not directly identify detoxification as being enriched exclusively in HR populations (Figure 7). Many of the same processes were enriched in both biotypes, which include chlorophyll and glucose catabolism, RNA metabolism, heme oxidation, phosphate transport, and proteolysis. However, these processes are fundamental to plant growth and serve as an antioxidant in some capacities.

Investigation at the individual gene level did identify genes previously implicated with herbicide resistance [50]. A key gene found on an eccDNA in our study exclusive to the two resistant populations (CC2R and CC5R) was GSTF1 (Glutathione S-Transferase Phi 1—ALOMY3G11302 [39]). This gene belongs to the GST gene family, which encodes enzymes involved in detoxifying herbicides by catalyzing the conjugation of glutathione to herbicide molecules, making them less toxic and more water-soluble for excretion or transport out of plant cells [51]. This gene has been associated with resistance to multiple classes of herbicides, including triazine and atrazine, indicating a role in multiple-herbicide resistance [51]. Alternate alleles for GSTF1 have been discovered where some alleles may confer higher herbicide resistance than others. Notably, the GSTF1 in this study is 39 amino acids shorter than previously reported isoforms [51]. The presence of this gene on an eccDNA with novel genetic variation in our study suggests a possible role in herbicide resistance and that eccDNA-based gene amplification and/or ultra-expression may also be critical factors to consider. Our study also identified GSTU2, OPR1, and GSTF2 as present on eccDNA, but not exclusively in HR populations (Table 4). These genes have also been implicated in herbicide resistance; however, these results may also suggest that, perhaps, detoxification may not fully support the explanation for the resistant phenotype observed in our HR populations.

The genomic mechanisms that contribute to the biogenesis of eccDNA are still not well understood. Some explanations include illegitimate replication (replication slippage), non-homologous end joining, homologous recombination, microhomology-mediated break-induced replication [61,62,63], and likely some organized or directed mechanisms, such as the *EPSPS* gene amplification that confers glyphosate resistance [12]. It is speculated that eccDNA can exist in multimeric forms and undergo recombination as a mechanism of compounded building into larger complex structures [1,19]. Analysis of eccDNA origins revealed eccDNA biogenesis on every chromosome with hotspots (high frequency of eccDNA formation) on chromosomes 1 and 7 (Figure 6). The identification of eccDNA originating from all over the genome is consistent with *A. palmeri* [1], as well as recent studies in mammalian cells [64,65]. These data provide further evidence that eccDNA biogenesis contributes to evolutionary innovation by contributing global genetic heterogeneity, plasticity, and a critical element to a plant’s trajectory to rapid adaptation. To further expand on this thought, we considered the concept of a two-speed genome recently proposed by eccDNA analysis of the rice blast fungal pathogen [66]. This concept considers that segments or regions within a genome evolve at different rates, and that, perhaps, this concept can correlate with genes found as genomic focal amplifications. The study considered genes found on eccDNA and their origin as within a gene-rich/poor region of the genome, in addition to proximity to repetitive elements, and found that genes on eccDNAs were in fact under faster evolutionary rates when compared with genes not found as eccDNA [66]. We examined this concept with eccDNA in this study and only found a statistically significant result when comparing the HR and HS populations together, suggesting alternate evolutionary pressures between the HR and HS populations presented here. Perhaps the selective pressure and adaptative signatures that correspond to herbicide resistance have broader implications genome-wide and further support mechanisms that provide resistance beyond metabolic genes. It has been proposed that plants that have rapidly evolved to a xenobiotic pressure also have a new disposition to withstand other abiotic stresses [67]. The compartmentalized genome architectures of the pathogens could also be organized in a way that these evolutionary signatures are more pronounced when compared with large plant genomes like blackgrass (3.2 Gb).

## 5. Conclusions

Our analysis provides evidence of evolutionary innovation and useful insights into the abundance, coding content, functional domains, and biogenesis patterns of eccDNA in various blackgrass populations, with a focus on the differences between herbicide-resistant and herbicide-sensitive populations. These findings demonstrate that the pernicious weed blackgrass (*A. myosuroides*) carries eccDNA with functional domains associated with genes previously reported to be associated with herbicide detoxification. As the eccDNA content is not identical between herbicide-resistant plants descending from different families exhibiting equivalent NTSR phenotypes, these differences may help to explain the polygenic enhanced metabolic resistance seen in these plants. It is interesting to note that, unlike EPSP synthase in Palmer amaranth, as blackgrass plants exhibiting sensitivity to herbicides also carry eccDNA with protein domains associated with herbicide detoxification, differences in eccDNA content cannot be fully responsible for the different phenotypes observed. Our data add blackgrass to the expanding list of organisms, including human cells, yeast, Drosophilia, and Palmer amaranth, and add to the mechanistic knowledge of how they confer herbicide resistance, alongside acting in stress adaptation and other physiological and pathological states.

## Figures and Tables

**Table 1:** eccDNA characterization of herbicide-resistant and -sensitive blackgrass populations.

Sample ID	# eccDNA	Mean Length	Length Range	# eccDNA with Gene	# eccDNA with tRNA	% eccDNA with CDS
CC2R	4812	7058	31–28,980	1031	53	21.43
CC2S	4233	6994	60–25,087	1010	77	23.86
CC5R	5288	6946	51–29,081	1145	74	21.65
CC5S	4332	7002	54–28,260	958	45	22.11
Peldon	4443	6868	36–26,368	962	59	21.65
Lola91	5426	6918	49–27,814	1153	70	21.25
Roth	5663	7040	51–27,090	1291	70	22.8

**Table 2:** Functional protein domains shared by all herbicide-resistant and herbicide-sensitive eccDNA samples with at least 30 counts in one set or that were only found in all HS or HR samples. N.D. designates those functional protein domains that were not detected in all the samples of that grouping.

Pfam Accession	Annotation	# eccDNA
PF04195	Putative gypsy type transposon	416
PF00361	Proton-conducting membrane transporter	348
PF00146	NADH dehydrogenase	148
PF00223	Photosystem I psaA/psaB protein	139
PF00499	NADH-ubiquinone/plastoquinone oxidoreductase chain 6	133
PF00420	NADH-ubiquinone/plastoquinone oxidoreductase chain 4L	112
PF13237	4Fe-4S dicluster domain	103
PF00124	Photosynthetic reaction center protein	93
PF01578	Cytochrome C assembly protein	91
PF00346	Respiratory-chain NADH dehydrogenase, 49 Kd subunit	87
PF00662	NADH-Ubiquinone oxidoreductase (complex I), chain 5 N-terminus	81
PF01010	NADH-dehyrogenase subunit F, TMs, (complex I) C-terminus	78
PF00421	Photosystem II protein	48
PF00623	RNA polymerase Rpb1, domain 2	42
PF01535	PPR repeat	41
PF00006	ATP synthase α/β family, nucleotide-binding domain	38
PF13456	Reverse transcriptase-like	36
PF00276	Ribosomal protein L23	32
PF13041	PPR repeat family	31
PF01824	MatK/TrnK amino terminal region	30

**Table 3:** Top twenty functional protein domains shared by herbicide-sensitive eccDNA samples.

Pfam Accession	Annotation	# eccDNA
PF04195	Putative gypsy type transposon	361
PF00361	Proton-conducting membrane transporter	208
PF00223	Photosystem I psaA/psaB protein	107
PF00146	NADH dehydrogenase	91
PF00499	NADH-ubiquinone/plastoquinone oxidoreductase chain 6	81
PF13237	4Fe-4S dicluster domain	65
PF00124	Photosynthetic reaction center protein	63
PF00346	Respiratory-chain NADH dehydrogenase, 49 Kd subunit	62
PF00662	NADH-Ubiquinone oxidoreductase (complex I), chain 5 N-terminus	56
PF01578	Cytochrome C assembly protein	55
PF01010	NADH-dehyrogenase subunit F, TMs, (complex I) C-terminus	51
PF00420	NADH-ubiquinone/plastoquinone oxidoreductase chain 4L	43
PF13456	Reverse transcriptase-like	36
PF00421	Photosystem II protein	35
PF01535	PPR repeat	35
PF00006	ATP synthase α/β family, nucleotide-binding domain	34
PF00069	Protein kinase domain	32
PF00646	F-box domain	24
PF02874	ATP synthase α/β family, β-barrel domain	24
PF03040	CemA family	24

**Table 4:** Homologous *A. myosuroides* candidate genes of predicted eccDNA.

Category	eccDNA Gene ID	Homologous Candidate Gene	Identity (%)	E-Value	Bit Score
Resistant	CC2R_00001032	GSTF1	95.434	4.95 × 10^−162^	436
	CC5R_00001033	GSTF1	31.25	1.07 × 10^−14^	64.7
	CC5R_00001372	GSTF1	55	2.22 × 10^−91^	257
	Lola91_00001333	GSTU2	61.364	1.70 × 10^−42^	128
	Lola91_00001340	GSTU2	38.009	1.69 × 10^−42^	133
	Peldon_00000910	OPR1	59.6	7.35 × 10^−108^	306
	Peldon_00000911	OPR1	64.646	6.10 × 10^−44^	137
Sensitive	CC2S_00001307	GSTU2	65.741	8.99 × 10^−53^	157
	CC5S_00001012	GSTF2	61.176	1.37 × 10^−33^	105
	Roth_00001016	GSTU2	31.429	2.42 × 10^−24^	85.1

**Table 5:** *A. myosuroides* genes located in the genomic region with high number of mapped eccDNA.

Chr	Start (Mb)	End (Mb)	# Herbicide-Resistant eccDNA	# Herbicide-Sensitive eccDNA	Genes Located	Functional Annotation	
1	251.0	251.5	83	48	ALOMY1G03512		
					ALOMY1G03513	PF00646	F-box domain
					ALOMY1G03514	PF02485	Core-2/I-Branching enzyme
					ALOMY1G03515		
					ALOMY1G03516	PF01288	7,8-dihydro-6-hydroxymethylpterin-pyrophosphokinase (HPPK)
					ALOMY1G03517	PF00809	Pterin binding enzyme
5	8.5	9.0	133	104	ALOMY5G31542	PF01015	Ribosomal S3Ae family
					ALOMY5G31543	PF03641	Possible lysine decarboxylase
					ALOMY5G31544		
					ALOMY5G31545	PF00294	pfkB family carbohydrate kinase
					ALOMY5G31546		
					ALOMY5G31547		
					ALOMY5G31548		
					ALOMY5G31549		
					ALOMY5G31550		
					ALOMY5G31551	PF00069	Protein kinase domain
					ALOMY5G31552	PF00069	Protein kinase domain
					ALOMY5G31553	PF00069	Protein kinase domain
					ALOMY5G31554		
					ALOMY5G31555		
5	45.0	45.5	82	55	ALOMY5G32133	PF06747	CHCH domain
					ALOMY5G32134		
					ALOMY5G32135		
					ALOMY5G32136		
					ALOMY5G32137		
					ALOMY5G32138	PF00179	Ubiquitin-conjugating enzyme
					ALOMY5G32139	PF00829	Ribosomal prokaryotic L21 protein
					ALOMY5G32140		
					ALOMY5G32141		
7	243.5	244.0	130	80	ALOMY7G40250	PF02493	MORN repeat
					ALOMY7G40251		
					ALOMY7G40252		
					ALOMY7G40253	PF00023	Ankyrin repeat
					ALOMY7G40254	PF01694	Rhomboid family
					ALOMY7G40255		
					ALOMY7G40256	PF12937	F-box-like
					ALOMY7G40257	PF00194	Eukaryotic-type carbonic anhydrase
					ALOMY7G40258		
					ALOMY7G40259	PF00153	Mitochondrial carrier protein

## Data Availability

All data presented in this study are publicly available at the Sequence Read Archive (SRA) in Genbank under Bioproject #PRJNA1017797 and sample accessions SAMN37408031-SAMN37408037.

## Supplementary Materials

The following supporting information can be downloaded at: https://www.mdpi.com/article/10.3390/genes14101905/s1, Table S1: Summary and characterization of eccDNAs detected in multiple-herbicide-resistant blackgrass populations; Table S2: Summary and characterization of eccDNAs detected in multiple-herbicide-sensitive blackgrass populations; Table S3: Venn diagram result summary for herbicide-resistant eccDNAs with annotation; Table S4: Venn diagram result summary for herbicide-sensitive eccDNAs with annotation; Table S5: Summary and characterization of eccDNAs detected in all samples; Table S6: Venn diagram result summary for eccDNAs with annotation among parents; Table S7: Venn diagram result summary for eccDNAs with annotation in CC2 populations; Table S8: Venn diagram result summary for eccDNAs with annotation in CC5 populations; Table S9: Gene ontology enrichment of all herbicide-resistant eccDNA genes classified as biological processes (BPs), cellular components (CCs), and molecular functions (MFs); Table S10: Gene ontology enrichment of all non-target site herbicide-susceptible eccDNAs genes classified as biological processes (BPs), cellular components (CCs), and molecular functions (MFs); Table S11: Counts of eccDNAs mapping to the blackgrass genome—500 kb; Table S12: Counts of eccDNAs mapping to the blackgrass genome—1 Mbp; Summary of eccDNA genes located in previously detected QTL regions associated with blackgrass herbicide resistance.
